# Imitation Precious Stones

**Published:** 1888-03

**Authors:** 


### IMITATION PRECIOUS STONES.

Though the appearance of artificial precious stones is now so exactly imitative of the genuine article as to render the judgment even of an expert frequently at fault, it is claimed that the test of hardness is still infallible. Thus, the beautiful French paste, from which such attractive imitation diamonds are made, is a kind of glass, with a mixture of oxide of lead, the more of the latter the brighter the stone, but also the softer, and this latter is the serious defect. But by careful selection of the ingredients, and skill and manipulation, the lustre, color, fire and water of the choicest stones are, to the eyes of the ordinary purchaser, fully repro duced ; there are a few delicacies of color that cannot be perfectly given, depending as they do on some undiscoverable peculiarities of molecular arrangement, and not on chemical composition—these however, not being apparent to the uninitiated. M. Sidot, however, a well-known French chemist, is reported to have nearly reproduced the peculiarities in question—including the dichroism of the sapphire—by means of a composition, of which the base is phosphate of lime ; and other chemists have produced rubies and sapphires having the same composition as the genuine stones, and almost as hard.

